# Implementing a multi-centre hypoglycaemia management surveillance model through a co-designed approach to develop actionable insights in the UK

**DOI:** 10.3389/frhs.2026.1862035

**Published:** 2026-09-24

**Authors:** Sam Sherratt-Mayhew, Aashritha Buchipudi, Charles Page, Amanda Ling Jie Yee, Kalyaani Persad, Aqeelah Khatoon, Mariam Idrissi, Amrit Rakkar, Sara Abdi Osman, Sanna Waheed, Faiz Ahmed, Alexandra Solomon, Rajeev Raghavan, Lakshmi Rengarajan, Ahmed Iqbal, Pratik Choudhary, Punith Kempegowda

**Affiliations:** 1 Birmingham Medical School, College of Medicine and Health, University of Birmingham, Birmingham, United Kingdom; 2 Russells Hall Hospital, Dudley, United Kingdom; 3 The Royal Wolverhampton NHS Trust, Wolverhampton, United Kingdom; 4 Walsall Manor Hospital, Walsall, United Kingdom; 5 Division of Clinical Medicine, School of Medicine and Population Health, University of Sheffield, Sheffield, United Kingdom; 6 Department of Population Health Sciences, George Davies Centre, University of Leicester, Leicester, United Kingdom; 7 Department of Applied Health Sciences, School of Health Sciences, College of Medicine and Health, University of Birmingham, Birmingham, United Kingdom; 8 Department of Diabetes and Endocrinology, Queen Elizabeth Hospital Birmingham, University Hospitals Birmingham NHS Foundation Trust, Birmingham, United Kingdom

**Keywords:** diabetes, guideline adherence, hypoglycaemia, multicentre, quality improvement, surveillance

## Abstract

**Introduction:**

Severe hypoglycaemia is associated with increased mortality. While considered a never event, severe hypoglycaemias continue occurring in people admitted to hospital. Inconsistent data collection and surveillance limits our ability to assess trends, identify risk factors and implement targeted interventions. This study aimed to (1) co-design a multicentre audit model for inpatient hypoglycaemia (2), evaluate its acceptability and feasibility and (3) analyse hypoglycaemia management to inform quality improvement initiatives.

**Methods:**

A hypoglycaemia audit system was co-designed with diabetes specialists, resident doctors and medical students, guided by ERIC study recommendations and the CFIR framework. Piloted at one site, it then expanded to ten hospitals, where feasibility and acceptability were evaluated using stakeholder feedback and system performance. The model was delivered via Plan-Do-Study-Act cycles and train-the-trainer methods. Data on demographics, precipitants, treatments, and outcomes of clinically significant hypoglycaemias in people with diabetes were analysed to identify risk factors and adherence to guidelines. End-user and expert feedback informed system validation.

**Results:**

The system was refined via stakeholder input and Plan-Do-Study-Act cycles to ensure usability before expanding to ten hospitals, where it achieved high acceptability. Over 11 months, 1,451 severe hypoglycaemias in 945 patients were recorded. Common precipitants included illness and missed meals. Management varied significantly between hospitals, e.g., glucagon use ranged from 2.6% (Hospital C) to 38.7% (Hospital E). Two hospitals within the same NHS trust showed differences in hypoglycaemia severity, treatment choices and mortality despite similar patient demographics.

**Conclusions:**

The co-designed multicentre digital evaluation model for in-hospital hypoglycaemia was successfully implemented with high acceptability and feasibility. Key findings highlight gaps in management, including suboptimal treatment adherence and high recurrence rates. Addressing these issues through real-time monitoring, targeted interventions and improved guideline adherence could enhance patient outcomes. This standardised model could address differences in healthcare regionally and nationally for other conditions.

## Introduction

Hypoglycaemia is a significant and potentially life-threatening complication of treating diabetes with far-reaching biological, psychological and social consequences (1). Severe hypoglycaemia—defined as any instance of low blood glucose that results in marked cognitive impairment requiring external assistance for recovery—is associated with increased morbidity, mortality and healthcare resource utilisation (2–4). Despite advances in diabetes management, 18% of hospitalised people with diabetes experience hypoglycaemic episodes, highlighting persistent gaps in inpatient care, with 26% of those with Type 1 diabetes experiencing a severe hypoglycaemia episode and 7.0% of those with Type 2 diabetes (5).

A critical barrier to improving hypoglycaemia management is the lack of an audit model to capture the underlying factors contributing to these episodes. Existing data collection frameworks, such as the National Diabetes Audit (NDISA), focus primarily on the frequency of inpatient hypoglycaemia but do not provide insights into the contextual and systemic contributors to these events (6). While many healthcare institutions have implemented local audit processes, these are often fragmented, lack interoperability, and fail to facilitate cross-centre collaboration for benchmarking and shared learning (7, 8). Without a structured, scalable, and comprehensive audit and feedback model model, opportunities for care standardisation and targeted interventions remain limited.

Implementation research highlights the importance of engaging frontline healthcare professionals in designing and executing quality improvement initiatives (9, 10). Medical students and resident doctors frequently lead data collection and interventions, making them key audit and implementation science stakeholders. Involving them in co-designing the audit and feedback model model fosters a sense of ownership, enhances engagement, and strengthens the sustainability of quality improvement efforts (11). Furthermore, co-designed audits and quality improvement projects (QIPs) can improve collaboration between different healthcare professionals, enhance efficiency, and drive meaningful changes in clinical practice (12, 13).

A promising example of a co-designed audit and feedback model model in diabetes management is the Digital Evaluation of Ketosis and Other Diabetic Emergencies (DEKODE) model, which standardised diabetic ketoacidosis (DKA) audits across multiple institutions, improving the understanding of variation in care associated with DKA management (14–16). We sought a similar co-designed approach for hypoglycaemia management. By integrating implementation science principles and leveraging frontline healthcare professionals' expertise, this study sought to establish a scalable and sustainable framework for collecting data for auditing hypoglycaemia management, ultimately enhancing patient safety and standardising care. This study's objectives included:

1. Co-design a multicentre audit and feedback model model with end-users and clinical experts to assess the quality of care provided during hypoglycaemia management.
2. Evaluate the feasibility, acceptability, validity and scalability of the audit and feedback model.
3. Describe the findings from the dataset generated, identifying key trends and areas for improvement and auditing in hypoglycaemia care and providing this information as quarterly feedback reports to participating centres.

## Materials and Methods

### Study design and setting

This prospective, multicentre observational study was conducted from October 2023 to December 2024 across ten hospitals (A–J) within five NHS Trusts in England, serving a diverse population of over two million. The study was approved by the informatics and ethics teams of the NHS trusts containing the participating centres.

### Development of the data collection tool

The study was designed following best practice recommendations from the National Institute for Health and Care Excellence (NICE) and the Healthcare Quality Improvement Partnership (HQIP) to ensure methodological rigour in developing a standardised quality improvement project (QIP) as described in Table 1 (17, 18).

**Table 1:** Assessment of DEKODE hypoglycaemia model in comparison to the clinical audit quality recommendations from the NICE and HQIP.

Criteria	Assessment Question	Source	Addressed in the Study
Audit objectives are clearly defined and aligned with departmental goals and confirming current good practice or improving current practice.	Are the objectives of the audit clearly defined?	Both	Yes, the objectives were clearly defined to audit hypoglycaemia management and identify existing gaps in auditing practices across multiple hospitals.
	Does the objective align with improving or confirming current clinical practices?	HQIP	Yes, the objective was to improve the management of hypoglycaemia by developing a standardised data collection tool and implementing it across multiple hospitals.
Involvement of relevant stakeholders, including resident doctors, consultants, and patients.	Are relevant stakeholders engaged in the audit process?	NICE	Yes, stakeholders, including clinical experts, resident doctors, medical students, and resident doctors, were involved in the design, testing, and implementation of the QIP system.
	Is there evidence of stakeholder meeting minutes and attendance records?	NICE	Yes, initial and final stakeholder meetings were held to define the scope, discuss findings, and finalise the data collection tool (mentioned in the co-designing section).
Formation of a multidisciplinary audit team.	Has a multidisciplinary audit team been formed with clearly defined roles and responsibilities?	NICE	Yes, a multidisciplinary team was formed, including consultants, resident doctors, and medical students, with defined roles for overseeing processes, data collection, and training.
Selection of audit criteria based on the best available evidence and clinical guidelines, reflecting critical aspects of patient care.	Are the audit criteria based on the best available evidence and clinical guidelines?	NICE	Yes, the criteria were based on IHSG Level 2 and Level 3 episodes, aligning with the International Hypoglycaemia Study's guidelines.
	Do the audit standards cover all key aspects of the clinical practice related to the audit's objective?	HQIP	Yes, the audit standards covered essential areas such as glucose levels, treatment protocols, and patient outcomes.
	Are the criteria relevant to critical aspects of patient care in diabetes and endocrinology?	NICE	Yes, the criteria were relevant to managing hypoglycaemia, a critical aspect of diabetes care.
	Are the measures (standards) sensitive and specific enough to accurately identify true positive and true negative cases?	HQIP	While the focus was on relevant hypoglycaemic episodes, the sensitivity and specificity of the standards are discussed and all steps to increase these values have been taken.
Comprehensive and reliable data collection plan, including sources, methods, and quality assurance.	Is there a comprehensive plan for data collection, including sources and methods?	NICE	Yes, the study involved developing a comprehensive data collection tool, a two-tiered data storage system, and training sessions for consistent data entry across hospitals.
	Are key terms defined, and instructions for decision-making specified?	HQIP	Yes, key parameters were defined, and categorical data were simplified to ensure consistent data collection.
	Are data collectors properly trained, and is inter-rater reliability tested to ensure consistency?	Both	Yes, training sessions were conducted for data collectors, and data entry was tested for formatting issues and gaps before full implementation.
	Was data collection pilot-tested before full implementation?	HQIP	Yes, pilot testing was conducted in Hospital A to identify and resolve formatting issues and gaps in the data fields.
Data sources are specified and capable of yielding quality data.	Are the data sources for each standard clearly specified?	HQIP	Yes, the data sources included hospital records of blood glucose levels, with specific focus on IHSG Level 2 and Level 3 episodes.
	Are the data sources likely to produce high-quality data efficiently?	HQIP	Yes, the data sources were considered reliable, and the system was designed to ensure efficient data collection across multiple hospitals.
Data quality is ensured through validation, accuracy, completeness, and adherence to protocols.	Are the data accurate, complete, and fit for purpose?	Both	Yes, regular validation checks were performed, and any anomalies were reviewed and corrected to ensure data accuracy.
	Is adherence to the case selection and data collection protocol monitored?	HQIP	Yes, adherence was ensured through training, regular validation checks, and monitoring by the audit team.
	Are there processes in place to prevent threats to data quality during collection and collation?	HQIP	Yes, a pseudonymisation system was used, and anomalies were flagged and reviewed to maintain data quality.
Development and implementation of an action plan based on audit findings.	Is there a written action plan with timelines and responsibilities?	NICE	Yes, the study included the development of a protocol for implementing the system across hospitals and assigning roles and responsibilities to ensure effective data collection.
	Is there evidence of effective implementation of recommended changes?	NICE	Yes, the data collection system was iteratively refined based on feedback and successfully implemented across ten hospitals.
	Are necessary resources allocated for implementing changes?	NICE	Yes, resources included the involvement of medical students, resident doctors, and consultants, as well as training and support resources for consistent implementation.
Continuous monitoring, follow-up audits, and dissemination of results.	Are follow-up audits conducted to ensure sustained improvement?	NICE	Follow-up audits are being planned to assess the impact of interventions.
	Are mechanisms established for continuous monitoring and feedback?	NICE	Yes, regular validation checks and feedback mechanisms were established, including quarterly feedback reports to participating centres.
	Are audit results regularly reported and disseminated to all stakeholders?	NICE	Yes, results were disseminated through structured quarterly feedback reports to stakeholders.
Provision of training and access to consultation and support for staff involved in audits.	Are training programs provided for staff involved in audits?	NICE	Yes, training programs were provided for medical students and resident doctors involved in the data collection process.
	Is there access to consultation and support from experienced audit supervisors?	NICE	Yes, the process involved regular team meetings and consultation with clinical experts to guide the audit process.
Promotion of a supportive culture and integration of audit activities with clinical governance.	Is there a culture that supports audit activities and continuous improvement?	NICE	Yes, the study created a collaborative culture involving stakeholders at multiple levels, and training ensured that new cohorts could sustain the audit activities.
	Are audit activities integrated with broader clinical governance frameworks?	NICE	Yes, the study's design and implementation processes align with clinical governance principles, though specific integration details are not explicitly mentioned.

A multidisciplinary panel of clinical experts in hypoglycaemia participated in a structured co-design workshop to define key data elements. The co-design process incorporated input from stakeholders across the multidisciplinary team, including endocrinologists, diabetes specialist clinicians, resident doctors, medical students, health informatics professionals and quality improvement leads. Stakeholders contributed to the identification and prioritisation of clinically relevant data elements, the practical design of the data collection workflow, and the usability of the electronic data collection form. Consideration was given to the feasibility of data collection within routine clinical workflows and the accessibility of the tool for staff with differing levels of experience. This part of the co-design process was led by resident doctors and medical students, the primary stakeholders involved in data collection and learning about the audit process. A draft data collection form was developed based on this co-design process and was piloted for feasibility and usability. A two-tiered data collection system was adopted to ensure both usability and data security:

- Local Storage: Patient-identifiable data remained securely stored on local hospital servers in Excel.
- Pseudonymised Collection: An online form with a user-friendly, multi-device interface was implemented to collect pseudonymised data for analysis, leveraging cloud-based architecture for accessibility while maintaining security (19).

The data collection framework was formalised as the DEKODE Hypoglycaemia Model. Data parameters were created to collect information on the demographics, precipitants, treatments, and outcomes of clinically significant hypoglycaemias.

### Pilot testing and iterative refinement

A pilot study was conducted at Hospital A, where a team of resident doctors and medical students led QIP registration and secured information governance clearance. Hypoglycaemia data was extracted from health informatics systems by the trust analytics department, who developed a Structured Query Language (SQL) query to extract all hypoglycaemic events at the centre (blood glucose < 4.0 mmol/L). This search initially identified over 20,000 episodes in one year. To enhance feasibility, data collection was refined to include only clinically significant hypoglycaemia (Level 2 and Level 3 episodes as defined by the International Hypoglycaemia Study Group (IHSG) in people living with diabetes (PLwD) who presented to or in the hospital and the SQL programme was adjusted accordingly (hypoglycaemia either with documented cognitive impairment and/or nadir glucose of <3.0 mmol/L) (4, 20). Level 1 is considered to have a nadir between 3.0 and 4.0 mmol/L without cognitive impairment. The original pilot began with 20 Level 1 hypoglycaemic events, tested by a single data collector to assess initial feasibility. These data were not included in the study and served as an initial proof-of-concept. Following this, the first 25 data entries of clinically significant hypoglycaemia were collected by three team members and analysed by the whole team. These data were included. All data fields were completed except for two with unknown ethnicity and 23 without documented MMSE scores. No data-entry errors were identified during review. Standardisation was achieved by utilising multiple choice options in the data collection form, with no issues with standardisation noted during the pilot.

The pilot incorporated an iterative Plan-Do-Study-Act (PDSA) cycle (21), optimising question sequencing for efficiency, refining terminology to reduce ambiguity, and integrating stakeholder feedback loops through regular engagement meetings where progress, feedback and action were monitored. Following three months of iterative refinement and regular analysis of inputted data, a consensus was reached on the final data collection form (shown in Supplementary Data Sheet 1).

### Scaling and implementation across sites

Post-pilot, the DEKODE Hypoglycaemia Model was expanded to five additional hospitals (B–F) to assess scalability and usability in real-world settings. Each site established a local implementation team comprising consultants (responsible for clinical governance and oversight), resident doctors and medical students (responsible for data collection and QIP approvals) and health informatics staff (assisting with data extraction).

A train-the-trainer model was employed, where the team from Hospital A provided structured training sessions for new staff at expansion sites based on their own experience and stakeholder input (22). These sessions included standardised teaching, using example patient data, to demonstrate how to identify eligible hypoglycaemic episodes and correctly complete the data collection form. Training was supplemented by written guidance on obtaining local quality improvement approval and requesting the relevant patient data lists. Regular drop-in sessions were also provided by members of the original pilot team and consultant clinicians to address queries, troubleshoot issues and support consistent implementation across participating centres. This approach ensured adherence to the protocol, mitigated knowledge loss due to staff turnover, and promoted a sense of ownership. To maintain data integrity and quality control, a validation team (statistician and clinical expert) conducted routine audits using automated anomaly detection to flag inconsistencies, manual data verification by independent researchers and an error resolution protocol of two researchers revalidating suspected errors.

A structured implementation protocol and workflow (Supplementary Data Sheet 2) guided the phased rollout to four additional hospitals (G–J) alongside the finalised data collection form. To promote sustainability, streamlined training resources and support mechanisms were embedded within the implementation framework. These elements were aligned with the four core constructs of Normalization Process Theory (NPT) to support the routine integration of this complex intervention into clinical practice at new centres and to facilitate onboarding new team members. Coherence was addressed by simplifying and refining the train-the-trainer model based on experiential learning. Cognitive participation was fostered through ongoing co-design and iterative revisions between end users and the research team. Collective action was supported through the integration of the intervention into existing workflows, facilitated by defined roles and resource allocation. Monitoring was sustained via ongoing team meetings that discussed continuous evaluation, shared learning and refinement of the implementation strategy (23).

## Data collection and analysis

### Data collection

The final multicentre dataset included all IHSG Level 2 and Level 3 hypoglycaemic episodes in individuals with diabetes aged ≥16 years from October 2023 to December 2024 across ten hospitals (A–J). As this is an audit investigating hypoglycaemia, hypoglycaemic episodes were chosen as the unit of analysis rather than patients. Whilst this does introduce the risk of clustering, it reduces the risk of selection bias by including recurrent episodes. As this audit seeks to assess and improve adherence to Joint British Diabetes Societies (JBDS) guidelines, this was deemed preferable to analysing patient-by-patient.

### Statistical analysis

An exploratory analysis of the collected audit data from the participating centres was conducted. Statistical analyses were conducted using Prism GraphPad v10.3.0 and R v4.4.1. As this project was designed as an audit and implementation study, no *a priori* power calculation was performed; all eligible data were collected. Normality of continuous data was assessed using the Shapiro–Wilk test. Non-normally distributed and ordinal data were described using medians and interquartile ranges (IQRs), and non-parametric hypothesis testing was performed using Mann–Whitney U tests. Normally distributed data were presented as means and standard deviations, and parametric hypothesis testing was performed using unpaired t-tests. Categorical variable data were reported as absolute counts (n) and proportions (x%), with Fisher's exact test used to determine significance. Significance was set at *p* < 0.05. Where anomalies or missing data were identified, data were cross-checked against original patient records from local centres by two researchers to ensure accuracy. As this study's primary aim was to co-design, evaluate and implement a surveillance system, adjusting for population differences, size and services provided and differences in intra-trust policy between centres was deemed to be beyond the scope of this manuscript. Multi-group comparison of data across all hospitals is therefore descriptive, with only two large, tertiary centres in the same trust being quantitatively compared.

### Feedback

Following multiple PDSA cycles of iterative refinement (21), a feedback form was sent to all team members to gauge the final acceptability and efficacy of the model amongst both end-users (medical students and resident doctors) and clinical experts. This will allow further refinement of the model in the future and homing in on important parameters. Questions were asked about the role of participants, aims of team members, data collection experiences, quality of the model, technical difficulties, how useful the project was to them and whether they made any implementations from the data. See Supplementary Data Sheet 3 for the exact questions utilised in the form. Likert scales from 0 to 10 (1 = poor, 10 = excellent) were used to gain qualitative insights into participants' opinions (24).

## Results

### Co-design a multicentre digital evaluation model to assess the quality of care provided during hypoglycaemia management

The DEKODE Hypoglycaemia model enabled the successful co-design and implementation of an audit and feedback model across 10 hospitals within five NHS trusts. This system was developed collaboratively with end-users who provided feedback throughout to allow for iterative refinement to ensure its practicality, user-friendliness and clinical relevance. Table 1 describes how we met the recommendations of HQIP and NICE (17, 18).

Multiple stages of the co-design, pilot and scaling of the digital evaluation model utilised interventions from the ERIC recommendations (25). A train-the-trainer model was used to disseminate knowledge to peers, requiring minimal senior input for the pilot study and keeping resource requirements low. We ensured regular training sessions were held both virtually and in-person concerning JBDS guidelines and IHSG criteria, to ensure end-users understood how to implement the system and its purpose (22, 26). This encouraged designation and leadership by promoting team members to leads of a participating centre. The co-design of educational materials for using the audit and feedback model ensured the training received was standardised, whilst local trainers could educate peers on the nuances of local cultures and health records.

### Acceptability and feasibility of this system across healthcare institutions

Feedback from medical students, resident doctors, information governance and ethics teams confirmed the system's feasibility and alignment with local policies. The pilot successfully assessed time burdens on the end-users and data completeness of hypoglycaemia records in hospital record systems. Input from end-users during this time allowed for co-design and iterative refinement. The internet-accessible data entry system allowed simultaneous input across locations, maintaining strict data security through a two-tier system.

21 members of the team, 17 end-users and four clinical experts, completed the feedback form assessing acceptability and validity. The anonymised responses are included in Supplementary Data Sheet 4. The feedback demonstrated the iterative refinement of the data collection form was successful in creating a clear form with a high level of organisation and flow, with a median Likert score of 10 (IQR 8−10) for clarity and 9 (7–10) for flow and organisation, showing acceptability amongst end-users and clinical experts. Both end-users and clinical experts reported learning more about hypoglycaemia from undertaking the project (7–10) and implementing change in their practice (7–10). The median number of reported data episodes collected was 100 per end-user (IQR 60−160), demonstrating feasibility amongst end-users. Finally, three clinical experts reported using data from the model to initiate local implementations for local guideline improvements.

### Adherence to consolidated framework for implementation research (CFIR) principles

#### Intervention characteristics

The audit and feedback model embodies key CFIR principles by emphasising adaptability and flexibility to integrate with diverse local health record systems and available datasets (a determinant that varied locally). Furthermore, the system aligns with CFIR's emphasis on equitable implementation, as its low-resource demands make it particularly suitable for use in resource-limited settings (27, 28). This may have extended application in low- and middle-income countries, where the burden of diabetes is disproportionately high. This adaptability served as an implementation strategy to address variations in technological infrastructure and data availability across participating centres. By avoiding dependence on a single data source or electronic platform, DEKODE reduced compatibility and resource barriers that might otherwise have limited adoption and facilitated implementation across heterogeneous healthcare settings.

#### Outer setting

There is increasing national and international drive to improve the quality and safety of diabetes care (e.g., NHS priorities, NICE guidelines, WHO diabetes targets). DEKODE aligns with these external drivers by offering a model that supports quality improvement, reporting and benchmarking, helping institutions comply with regulatory standards and funding expectations. Regular feedback reports that match local data with pooled data from all participating centres influence peer practice, thereby increasing implementation uptake (27). The provision of centre-specific reports alongside pooled data therefore represented an implementation strategy responsive to these external pressures, translating national quality and safety priorities into locally actionable information. This feedback and benchmarking mechanism provided participating teams with an opportunity to identify areas for improvement relative to other centres, thereby reinforcing engagement and supporting continued adoption of the audit and feedback model.

#### Inner setting

The audit and feedback model was designed with common barriers to QIPs within the NHS in mind. By engaging medical students and resident doctors in data collection, the model aligned with their mandatory involvement in audits or QIPs as part of their training. Case identification was flexible to accommodate the varying resources and infrastructures of participating hospitals, a local determinant varying centre to centre. In centres with electronic health records, cases were identified through established electronic infrastructure. Conversely, in smaller district general hospitals such as Hospital J, where documentation remains paper-based, case identification relied on diabetes specialist nurses receiving referrals for patient review, which is a mandatory step for any serious episode of hypoglycaemia, a determinant relating to infrastructure. This adaptability supported successful implementation across diverse operational contexts, reinforcing the model's responsiveness to the structural and cultural characteristics of each inner setting (27). These flexible case-identification strategies were therefore deliberately matched to the infrastructure of each participating centre, addressing differences in information systems and staffing capacity while maintaining a common surveillance framework. Embedding data collection within existing audit and training structures further reduced the organisational burden of implementation and facilitated integration into routine clinical practice.

#### Characteristics of individuals

DEKODE's role in enhancing healthcare professionals' knowledge and skills aligns with CFIR's emphasis on individual attributes and learning, which improve implementation success. By utilising a train-the-trainer model, self-efficacy was promoted, and a sense of ownership of the implementation was fostered, increasing knowledge and belief in the system (27). The train-the-trainer approach therefore served as an implementation strategy targeting individual-level determinants, particularly knowledge, confidence and perceived capability to undertake surveillance activities. By developing local expertise rather than relying on a central research team, this approach also promoted ownership and supported the sustainability of implementation beyond the initial training period.

### Identifying key trends and areas for improvement in hypoglycaemia care

A total of 2,412 data entries were collected over 11 months, documenting 1,451 episodes of level 2 or 3 hypoglycaemia across 945 PLwD (714 episodes were excluded due to being IHSG Level 1 and 247 were excluded as they occurred in someone who was not a PLwD). The median age of the patient was 72 years, female: male ratio - 1:1.1. 72.6% of PLwD had type 2 diabetes. Of the episodes, intercurrent illnesses (42.1%) and fasting/missing meals (39.8%) were the most common precipitating factors. Oral glucose (31.4%), IV dextrose (37.0%), and glucagon (12.3%) were the most common treatments used to address hypoglycaemia. See Table 2 for a summary of the dataset. Other than ethnicity and degree of cognitive impairment, no other parameters were found to have missing data (missing data is displayed in Tables 2, 3). Of note, Hospital I is an outpatient and community rehabilitation centre, hence only one clinically significant hypoglycaemia was identified. Hospital J had not completed data collection at the time of manuscript submission.

**Table 2:** Patient demographics.

Parameter	Category/Value	Overall	A	B	C	D	E	F	G	H	I	J
Cohort		945	205 (21.7)	63 (6.7)	119 (12.6)	123 (13.0)	255 (27.0)	142 (15.0)	7 (0.7)	18 (1.9)	1 (0.1)	12 (1.3)
Sex	Female	451 (47.7)	85 (41.5)	38 (60.3)	52 (43.7)	66 (53.7)	129 (50.6)	64 (45.1)	5 (71.4)	6 (33.3)	0	6 (50.0)
	Male	494 (52.3)	120 (58.5)	25 (39.7)	67 (56.3)	57 (46.3)	126 (49.4)	78 (54.9)	2 (28.6)	12 (66.7)	1 (100.0)	6 (50.0)
	F:M ratio	1:1.10	1:1.41	1.52:1	1:1.28	1.16:1	1.02:1	1:1.22	2.5:1	1:2	N/A	1:1
Ethnicity	White	635 (67.2)	142 (69.3)	13 (20.6)	92 (77.3)	70 (56.9)	179 (70.2)	116 (81.7)	3 (42.9)	11 (61.1)	0	9 (75.0)
	Asian	165 (17.5)	44 (21.5)	35 (55.6)	7 (5.9)	27 (22.0)	38 (14.9)	7 (4.9)	3 (42.9)	1 (5.6)	1 (100.0)	2 (16.7)
	Black	73 (7.7)	9 (4.4)	8 (12.7)	8 (6.7)	13 (10.6)	27 (10.6)	4 (2.8)	1 (14.3)	2 (11.1)	0	1 (8.3)
	Other/mixed	15 (1.6)	6 (2.9)	6 (9.5)	0	1 (0.8)	2 (0.8)	0	0	0	0	0
	Unknown	57 (6.0)	4 (2.0)	1 (1.6)	12 (10.1)	12 (9.8)	9 (3.5)	15 (10.6)	0	4 (22.2)	0	0
Age	Years, Median(IQR)	72 (59−82)	63 (50−75)	71 (58−83)	75 (59−84)	68 (59−78)	73 (59−83)	75 (63−82)	82 (73−85)	82 (75−86)	87 (87−87)	77 (58–81)
Diabetes Type	1	248 (26.2)	49 (23.9)	11 (17.5)	40 (33.6)	31 (25.2)	73 (28.6)	36 (25.4)	2 (28.6)	3 (16.7)	0 (0.0)	3 (25.0)
	2	686 (72.6)	151 (73.7)	52 (82.5)	78 (65.5)	89 (72.4)	180 (70.6)	106 (74.6)	5 (71.4)	15 (83.3)	1 (100.0)	9 (75.0)
	Other type	11 (1.2)	5 (2.4)	0	1 (0.8)	3 (2.4)	2 (0.8)	0	0	0	0	0

All results by hospital. All results given as *n* (%), where *n* represents the number of patients. Age given as median (IQR) in years.

**Table 3:** Comparison of results by episode between hospitals A and D, two similar centres in the same NHS trust.

Parameter	Category/Value	A	D	*P* value
Cohort		361	147	
Sex	Female	182 (50.4)	87 (59.2)	
	Male	179 (49.6)	60 (40.8)	
Ethnicity	White	230 (63.7)	92 (62.6)	
	Asian	78 (21.6)	27 (18.4)	
	Black	39 (10.8)	15 (10.2)	
	Other/mixed	6 (1.7)	1 (0.7)	
	Unknown	8 (2.2)	12 (8.2)	
Age	Years, Median (IQR)	63 (50–75)	68 (59–78)	0.0118
Diabetes Type	1	115 (31.9)	42 (28.6)	
	2	240 (66.5)	100 (68.0)	
	Other type	6 (1.7)	5 (3.4)	
Antidiabetic medications	Insulin	272 (75.3)	98 (66.7)	0.0485
	Biguanides	99 (27.4)	46 (31.3)	
	Sulfonylureas	26 (7.2)	14 (9.5)	
	DPP4I	46 (12.7)	18 (12.2)	
	SGLT2	21 (5.8)	5 (3.4)	
	GLP-1	2 (0.6)	0	
	Thiazolidinediones	0	0	
	Antipsychotics	18 (5.0)	4 (2.7)	
IHSG Event level	2	265 (73.4)	88 (59.9)	0.004
	3	96 (26.6)	59 (40.1)	
Cognitive impairment^a^	Yes	146 (56.8)	69 (63.9)	
	No	111 (43.2)	39 (36.1)	
MMSE score^a^	0–10	10 (90.9)	0	
	10–20	1 (9.1)	0	
	20–30	0	0	
Precipitant	Fasting/missed meals	203 (56.2)	33 (22.4)	<0.0001
	Intercurrent illness	113 (31.3)	96 (65.3)	<0.0001
	Incorrect medication dose	31 (8.6)	11 (7.5)	
	Exercise	0	0	
	Unclear	78 (21.6)	30 (20.4)	
	Multiple causes	57 (15.8)	18 (12.2)	
	Single cause	304 (84.2)	129 (87.8)	
Furthest line treatment reached	Not fully documented	50 (13.9)	27 (18.4)	
	Oral glucose	97 (26.9)	23 (15.6)	0.0079
	IV Dextrose	198 (54.8)	87 (59.2)	
	Glucagon	16 (4.4)	10 (6.8)	
Total number of episodes where treatment was used^a^	Oral glucose	212 (73.6)	61 (55.0)	0.0005
	Dextrose	211 (58.6)	97 (66.0)	
	Glucagon	16 (3.4)	10 (5.7)	
Hyperglycaemia	Yes	5 (1.4)	7 (4.8)	0.0459
	No	356 (98.6)	140 (95.2)	
Episode Outcome	Admitted to hospital	4 (1.1)	2 (1.4)	
	Care continued as inpatient	332 (92.0)	120 (81.6)	0.0015
	Sent to ITU	6 (1.7)	5 (3.4)	
	Sent home	1 (0.3)	1 (0.7)	
	Death	18 (5.0)	19 (12.9)	0.004
Discharge outcome	Died	87 (24.1)	51 (34.7)	0.0206
	Complex	165 (45.7)	53 (36.1)	0.0486
	Simple	87 (24.1)	32 (21.8)	
	Other	22 (6.1)	11 (7.5)	
Adjustment	Medication change	128 (35.5)	74 (50.3)	0.0026
	Specialist team referral	65 (18.0)	46 (31.3)	0.0014
	Palliative care plan	86 (23.8)	30 (20.4)	
	CGM started	10 (2.8)	1 (0.7)	
	Glucagon prescribed	1 (0.3)	0	
Medication change	Stop sulfonylureas	5 (3.9)	9 (12.2)	0.041
	Reduce sulfonylureas	7 (5.5)	3 (4.1)	
	Stop insulin	16 (12.5)	18 (24.3)	0.0496
	Reduce insulin	97 (75.8)	39 (52.7)	0.001
	Changes to others	24 (18.8)	18 (24.3)	

All results given as *n* (%), where *n* represents the number of episodes. Age given as median (IQR). Where *p* values are not given there was no significant difference.

a Excluding unknowns not listed on hospital records. 350 episodes from Hospital A and all episodes from Hospital D did not record MMSE.

### Variations across hospitals

Large differences were observed in the management and outcomes of hypoglycaemic episodes between hospitals (see Table 2). Notably, Hospital C used glucagon in only 2.6% of episodes, while Hospital E used it in 38.7% of episodes, highlighting differences in management approach.

### Comparing similar hospitals

Analysis of two comparable hospitals (A and D) within the same NHS trust revealed significant differences in treatment approaches and outcomes despite similar patient demographics (Table 3). These hospitals were comparable as they are both large centres serving similar patient populations in Birmingham, UK, with over 700 beds each; each offers a variety of tertiary services and is part of the same NHS trust with the same intra-trust guidelines. Hospital A used more oral glucose (*p* = 0.0079), while Hospital D had higher mortality rates following significant episodes (*p* = 0.0206). Therapeutic adjustments, including insulin dose reductions and specialist team referrals, also varied significantly between the two hospitals. Of note, as this is an audit project collecting all applicable significant hypoglycaemic events, some events occurred in the same patient and are non-independent. The median number of episodes per patient in the dataset was 1 episode (IQR 1-1), the mean number of episodes per patient was 1.5 episodes and 169 of the 945 patients had more than one episode.

### Challenges and solutions

Challenges related to participation and delays due to clinical duties were overcome through strategic recruitment, regular team meetings, and ongoing communication with information governance and ethics teams. These efforts ensured the system was feasible to implement in busy clinical environments and could be scaled further to additional centres.

### Scalability and sustainability

The audit and feedback model was scaled across ten hospitals, supported by developing a structured implementation guide to facilitate onboarding new centres into the model. Sustainability was ensured through the recruitment of new cohorts of medical students and resident doctors at the beginning of each academic year. This cyclical integration of trainees ensured the continuity of data collection and maintained engagement with the initiative over time, as reflected in the final feedback from the DEKODE team. The first series of quarterly feedback forms were delivered to participating centres with both local and regional data (see Supplementary Data Sheet 5, the quarterly feedback form for Hospital J). This approach can sustain follow-up audit cycles indefinitely, ensuring ongoing monitoring and improvement in hypoglycaemia care.

### Illustrative use case

The following use case provides a step-by-step description of how to replicate this audit and feedback approach at a new centre. First, an aspiring centre lead identifies a suitable team of medical students and/or resident doctors, along with supervising consultant(s). The local clinical audit department is then contacted by the centre lead to obtain the appropriate audit registration and governance approvals, while the informatics team generates a list of all eligible cases, defined as hypoglycaemic episodes with a nadir glucose <3.0 mmol/L. The identified cases are subsequently reviewed by the trained data-collection team using the data-collection tool. The predefined parameters to be recorded for each episode are provided in Supplementary Data Sheet 1.

Before commencing full data collection, supervisors and data collectors undergo training in using the tool. Data collection initially begins with a small pilot sample. The pilot dataset is then analysed and independently checked by the data collectors, supervisor and statistician to assess the feasibility, clarity and quality of data collection. Findings from the pilot are used to refine the data collection process and inform a train-the-trainer approach, enabling additional members of the local team to be trained and participate in the surveillance activity. Once data collection is complete, adherence to JBDS and relevant local hypoglycaemia management guidelines can be assessed, providing a basis for identifying areas for improvement and informing locally relevant interventions.

## Discussion

The DEKODE Hypoglycaemia model enabled the co-design and implementation of an audit and feedback system across ten NHS hospitals. Developed with end-users, the system was iteratively refined and aligned with HQIP, NICE and JBDS standards. Using a train-the-trainer approach, the model promoted scalability, self-efficacy and sustainability through the annual recruitment of trainees. Grounded in CFIR principles, DEKODE supports equitable implementation and continuous quality improvement in diabetes care across diverse healthcare settings. Despite varied local infrastructures, it demonstrated adaptability and feasibility, collecting over 2,400 data entries in 11 months. Analysis revealed inter-hospital variation in hypoglycaemia care, identifying key areas for improvement. This has enabled targeted interventions to improve hypoglycaemia care. Furthermore, the active involvement of medical students and resident doctors in data collection and implementation fostered critical thinking, problem-solving, and leadership skills, aligning with recommendations for engaging trainees in audit and quality improvement projects (29, 30).

Existing studies have primarily examined isolated causes of hypoglycaemia and lacked the potential to be a standardised framework for comprehensive data collection across multiple centres (31–33). Our audit and feedback model addresses this gap by establishing a robust, scalable system adaptable across diverse healthcare settings.

Our study's co-design process is a key strength, which engages stakeholders and end-users to ensure adaptability across hospitals with varying resources and medical record-keeping systems. Additionally, the system's minimal resource requirements—only necessitating an internet connection and trained end-users—enhance its global applicability, potentially meaning the system could be used in low- and middle-income countries where IT infrastructure may be limited, although this still may prove difficult as an internet connection is still required (34). In developed nations, where EHR standardisation remains an ongoing challenge, DEKODE can help identify trends in hypoglycaemia care without requiring EHRs (35).

The sensitivity and specificity of the collected data depend on the accuracy of clinical documentation and end-users' ability to extract relevant information from health records. While training and standardisation efforts have mitigated these challenges, our current protocol does not resolve this limitation. Additionally, incomplete integration between separate systems—particularly for emergency admissions and community-based events—may impact data completeness and limit generalisability. However, ongoing NHS initiatives toward integrated EHRs are expected to help reduce this issue soon (36).

### Future directions

We plan to continue implementing quarterly feedback summaries for participating centres, providing insights into hypoglycaemic event patterns, patient outcomes, and therapeutic adjustments. These summaries will inform continuous improvement efforts and structured educational interventions, which have been shown to reduce hypoglycaemic episodes (37–39).

Next steps include automating data quality screening and feedback mechanisms, enabling hospitals to analyse local trends independently. Quantitative analysis of quality assurance investigating reliability and detected data entry errors could be completed on completion of data collection alongside the piloting process detailed. Future work exploring differences between centres should consider controlling for clustering. Ultimately, this scalable methodology could be adopted globally, similar to the DEKODE diabetic ketoacidosis model, and be integrated into EHR systems with a condensed set of impactful clinical parameters (15).

## Conclusion

We co-designed a scalable, multicentre hypoglycaemia care audit and feedback model, with good acceptability and sustainability. Findings revealed high-risk demographics, key precipitants and care gaps, guiding future improvements. Further refinement, including automation, is currently underway to enhance usability and long-term sustainability.

## Data Availability

The original contributions presented in the study are included in the article/Supplementary Material, further inquiries can be directed to the corresponding author.
