# Supplementary material for: Implementing a multi-centre hypoglycaemia management surveillance model through a co-designed approach to develop actionable insights in the UK
Source: Front Health Serv. 2026 Sep 24;6:1862035. doi: 10.3389/frhs.2026.1862035 (PMC13646156; doi:10.3389/frhs.2026.1862035)
Supplement: Supplementary file 1 [file Datasheet1.pdf]

Patient details

[redacted] Switch accounts

Not shared

* Indicates required question

Name of the person collecting the data (your name) *

Your answer

What was the lowest blood glucose level (in mmol/L) during this hypoglycaemic episode? *

Your answer

Which Hospital was the patient admitted to? *

- City Hospital, Birmingham
- Good Hope Hospital
- Heartlands Hospital
- New Cross Hospital, Wolverhampton
- Queen Elizabeth Hospital Birmingham
- Russells Hall Hospital, Dudley
- Sandwell Hospital
- Walsall Manor Hospital
- Hereford County Hospital
- Other:

Patient identification number (this can be anything that you generate for your reference to identify this patient in the future. Eg: QEHB-0001 for Queen Elizabeth Hospital Birmingham patient no. 1) *

Your answer

Year of Birth *

Your answer

Gender *

- Male
- Female

Ethnicity *

- White – British
- White – Irish
- Other white background
- Black or Black British-Caribbean
- Black or Black British-African
- Other Black Background
- Asian or Asian British-Indian
- Asian or Asian British-Pakistani
- Asian or Asian British-Bangladeshi
- Chinese
- Other Asian Background
- Mixed – White and Black Caribbean
- Mixed – White and Black-African
- Other Mixed Background
- Other Ethnic background
- Not Known
- Other:

What type of diabetes? *

- ◯ 1
- ◯ 2
- ◯ 3c
- ◯ Gestational diabetes
- ◯ No history of diabetes
- ◯ Other:

Charlson Comorbidity Index (CCI) prior to hospitalisation (please enter 999 if unable to accurately calculate this) *

Use this formula to calculate CCI:
https://www.mdcalc.com/calc/3917/charlson-comorbidity-index-cci#use-cases

Your answer

Weight in kg (enter 999 if not available) *

Your answer

Height in cm (enter 999 if not available) *

Your answer

Most recent HbA1c in mmol/mol (prior to or upto on the day of hospitalisation) (enter 200 if not available) *

Your answer

Date (enter 09.09.1999 if not known) and time (enter 09:09 if not known) when HbA1c was last checked before admission *

Date

dd/mm/yyyy

Time

:

Next

Clear form

Never submit passwords through Google Forms.

This content is neither created nor endorsed by Google. Report Abuse - Terms of Service - Privacy Policy

Google Forms

Patient details

[redacted] Switch accounts

Not shared

* Indicates required question

Medication details

Was your patient treated with insulin for diabetes before admission with this hypoglycaemic episode? *

- Yes
- No

Back Next Clear form

Never submit passwords through Google Forms.

This content is neither created nor endorsed by Google. Report Abuse - Terms of Service - Privacy Policy

Google Forms

Patient details

[redacted] Switch accounts

Not shared

* Indicates required question

Other medications for diabetes

Select all medications the patient was on before admission for treating diabetes? *

- [ ] None
- [ ] sulfonylureas
- [ ] meglitinides
- [ ] biguanides
- [ ] thiazolidinediones
- [ ] alpha-glucosidase
- [ ] GLP1 analogues
- [ ] DPP4 inhibitors
- [ ] SGLT2 inhibitors
- [ ] Other:

Back   Next   Clear form

Never submit passwords through Google Forms.

This content is neither created nor endorsed by Google. Report Abuse - Terms of Service - Privacy Policy

Google Forms

Patient details

[redacted] Switch accounts

Not shared

Draft saved

* Indicates required question

Antipsychotics

Was patient on any antipsychotics before presentation? *

- ○ Yes
- ◉ No

Back Next Clear form

Never submit passwords through Google Forms.

This content is neither created nor endorsed by Google. Report Abuse - Terms of Service - Privacy Policy

Google Forms

Patient details

[redacted] Switch accounts

Not shared

* Indicates required question

Episode details

Date (enter 09.09.1999 if not known) and time (enter 09:09 if not known) of hypoglycaemia *

Date

dd/mm/yyyy

Time

:

Method for blood glucose measurement during this hypoglycaemic episode

- Finger prick
- ABG
- Continuous glucose monitor
- Other:

Where was the patient at the time of hypoglycaemia? *

- ◯ Own home
- ◯ Care home
- ◯ Nursing Home
- ◯ Homeless
- ◯ Other hospital
- ◯ Admitted in the hospital during this episode
- ◯ Other:

Back Next Clear form

Never submit passwords through Google Forms.

This content is neither created nor endorsed by Google. Report Abuse - Terms of Service - Privacy Policy

Google Forms

What was patients clinical frailty score *

1. Very Fit: People who are robust, active, energetic, and motivated. These people commonly exercise regularly. They are among the fittest for their age.
2. Well: People who have no severe disease symptoms but are less fit than category 1. They exercise or are very active occasionally, e.g., seasonally.
3. Managing Well: People whose medical problems are well-controlled but are not regularly active beyond routine walking.
4. Living With Very Mild Frailty: Previously named "Vulnerable," While not dependent on others for daily help, symptoms often limit activities. A common complaint is being "slowed-up" and being tired during the day.
5. Living with Mild Frailty: These people usually have more evident slowing and need help in higher-order instrumental activities of daily living (IADLs) such as finance, transportation, heavy housework, and medication management. Typically, mild frailty progressively impairs shopping and walking outside alone, meal preparation, and housekeeping.
6. Living With Moderate Frailty: People need help with all outside activities and housekeeping. Inside often have problems with stairs, need help with bathing, and may need minimal assistance with dressing.
7. Living With Severe Frailty: Completely dependent for cognitive and physical personal care. However, they seem stable and not at high risk of dying (within six months).
8. Living with Very Severe Frailty: Completely dependent for personal care and approaching end of life. Typically they could not recover even from minor illnesses.
9. Terminally Ill: Approaching the end of life. This category applies to people with a life expectancy of under six months who are not otherwise living with severe frailty. (Many terminally ill people can still exercise until very close to death.)

- ○ 1
- ○ 2
- ○ 3
- ○ 4
- ○ 5
- ○ 6
- ○ 7
- ○ 8
- ○ 9

Back Next Clear form

Never submit passwords through Google Forms.

Patient details

[redacted] Switch accounts

Not shared

* Indicates required question

Outcome for the patient

Did the patient die during this episode? *

- Yes
- No

Back

Next

Clear form

Never submit passwords through Google Forms.

This content is neither created nor endorsed by Google. Report Abuse - Terms of Service - Privacy Policy

Google Forms

Patient details

[redacted] Switch accounts

Not shared

* Indicates required question

Hypoglycaemia episode Details

Level of Hypoglycaemia (IHSG definition) *

- Level 1, alert level (<3.9mmol/L but > 3mmol/L)
- Level 2, clinically significant (<3mmol/L
- Level3, Severe (Treatment requires the assistance of another person)

Precipitating cause of hypoglycaemia *

- Fasting/missed meals
- Incorrect dose of oral hypoglycaemic drugs
- Incorrect dose of Insulin
- Intercurrent illness
- Acute renal impairment
- Exercise
- Unclear
- Other:

Did the patient receive Glucagon from Home/Ambulance/hospital during the episode? *

- ◯ Yes
- ◯ No
- ◯ Unknown

Did the patient receive a glucose tablet/ glucose juice/ any other form of glucose oral substitution from Home/Ambulance/hospital during the episode?

- ◯ Yes
- ◯ No
- ◯ Unknown

Outcome of episode *

- ◯ Admission to ITU
- ◯ Admission to Hospital/ Care continued as already an inpatient
- ◯ Sent home from ER

Did patient have cognitive impairment during episode *

- ◯ Yes
- ◯ No
- ◯ Unkown

Mini Mental State Examination Score *

- ◯ 0-10
- ◯ 10-20
- ◯ 20-35
- ◯ 25-30
- ◯ Unkown

Creatinine at presentation (micromol/L) (enter 999 if not available) *

Your answer

eGFR at presentation (ml/min/1.73m2)) (enter 999 if not available) *

Your answer

What was the highest concentration of Dextrose given to the patient as part of hypoglycaemia management *

- ◯ 5%
- ◯ 10%
- ◯ 20%
- ◯ 50%
- ◯ Did not recieve Dextrose
- ◯ Other:

How much volume of Dextrose did the patient receive (enter values in ml. eg. 500). *

Your answer

How does the patient monitor sugar at home *

- ○ Cappilary Blood Glucose Test
- ○ Continous Glucose Monitor
- ○ Patient does not monitor blood glucose at home

Back Next Clear form

Never submit passwords through Google Forms.

This content is neither created nor endorsed by Google. Report Abuse - Terms of Service - Privacy Policy

Google Forms

Patient details

[redacted] Switch accounts

Not shared

* Indicates required question

Post correctional Hyperglycaemia

Did patient develop hyperglycaemia (glucose >20 mmol/L for >6 hours) after correction *

- Yes
- No

Back Next Clear form

Never submit passwords through Google Forms.

This content is neither created nor endorsed by Google. Report Abuse - Terms of Service - Privacy Policy

Google Forms

Patient details

[redacted] Switch accounts

Not shared

* Indicates required question

Change in therapy

What change was made in therapy on discharge to prevent further episodes *

	Yes	No
Stopped Sulfonylurea drugs (eg. Gliclazide)	◯	◯
Redcued Sulfonylurea drugs (eg. Gliclazide)	◯	◯
Insulin stopped	◯	◯
Insulin dose reduced	◯	◯
CGM (eg. Libre, dexcom) started	◯	◯
Referral to specialist team	◯	◯
Changes to other anti-diabetes medicines	◯	◯

Was glucagon prescribed on discharge? *

◯ Yes

◯ No

Back Next Clear form

Never submit passwords through Google Forms.

This content is neither created nor endorsed by Google. Report Abuse - Terms of Service - Privacy Policy

Google Forms

Patient details

[redacted] Switch accounts

Not shared

* Indicates required question

Discharge details

Date (enter 09.09.1999 if not known) and time (enter 09:09 if not known) of discharge *

Date

dd/mm/yyyy

Time

:

This is a required question

Back

Next

Clear form

Never submit passwords through Google Forms.

This content is neither created nor endorsed by Google. Report Abuse - Terms of Service - Privacy Policy

Google Forms

Discharge type *

Simple discharge	Complex discharge	Rapid discharge
• Involve minimal disturbance to the patient's daily routines.<br>• Does not prevent or hamper the patient being discharged to their usual place of residence.<br>• Will not require a significant change in support offered to the patient or their carer.<br>• Self-caring patient with no decline in functional ability as a result of illness.	• Deviates from the normal discharge pathway and requires complex coordination of services to enable safe discharge.<br>• This may include social work referrals, multidisciplinary meetings, continuing care checklists and a possible change between admission and discharge destination.	• May be simple or complex and is usually as a result of the end of life pathway or palliative discharge.
**E.g.**<br>• Pregnant woman with newly diagnosed diabetes<br>• Post-operative surgery	**E.g.**<br>• Frail elderly patient<br>• Patient with a mental illness<br>• Patient with learning difficulties<br>• Homeless person<br>• Person post-limb amputation<br>• Person requiring multi-agency support<br>• 'Vulnerable adult'<br>• Person with dementia or cognitive impairment	**E.g.**<br>• Person with terminal illness<br>• Transfer to a hospice<br>• Transfer to another intermediate care facility

- ○ Simple discharge
- ○ Complex discharge
- ○ Rapid discharge
- ○ Discharge against medical advice
- ○ Patient died during this hospital admission
- ○ Discharge to another hospital
- ○ Other:

Back Next Clear form

Patient details

[redacted] Switch accounts

Not shared

* Indicates required question

Discharge destination

Discharge destination *

- Home
- Care home
- Homeless
- Died
- Another hospital
- Other:

Was the patient on palliative care during admission or on discharge? *

- Yes
- No
- Unclear

Back Next Clear form

Never submit passwords through Google Forms.

This content is neither created nor endorsed by Google. Report Abuse - Terms of Service - Privacy Policy

Google Forms

Patient details

[redacted] Switch accounts

Not shared

Additional comments

Do you have any additional comments for this episode

Your answer

Back Submit Clear form

Never submit passwords through Google Forms.

This content is neither created nor endorsed by Google. Report Abuse - Terms of Service - Privacy Policy

Google Forms
