# Supplementary material for: Implementing a multi-centre hypoglycaemia management surveillance model through a co-designed approach to develop actionable insights in the UK
Source: Front Health Serv. 2026 Sep 24;6:1862035. doi: 10.3389/frhs.2026.1862035 (PMC13646156; doi:10.3389/frhs.2026.1862035)
Supplement: Supplementary file 2 [file Datasheet2.pdf]

DEKODE-Hypoglycaemia Participant Involvement Questionnaire

Thank you for participating in the DEKODE-Hypoglycaemia project. This questionnaire aims to understand your experience and any challenges encountered during data collection. Your feedback will help us improve our surveillance system. Estimated completion time: 5 minutes.

* Required

1. Full name *

Enter your answer

2. Email address *

Please enter an email

3. Hospital *

4. Approx date of start in DEKODE Hypoglycaemia project *

5. Approx date of end in DEKODE Hypoglycaemia project (add today's date if you are still actively involved in the project) *

6. Role in DEKODE-Hypoglycaemia (select all that apply) *

- [ ] Audit/QIP registration
- [ ] Data collection
- [ ] Data analysis
- [ ] Consultant Lead
- [ ] Other

7. How did you hear about DEKODE-Hypoglycaemia? *

- [ ] Word of mouth from colleague
- [ ] Social media
- [ ] DEKODE team
- [ ] SIMBA
- [ ] Comics
- [ ] Other

8. Why did you participate in DEKODE-Hypoglycaemia? (select all that apply) *

- [ ] Research experience and portfolio development
- [ ] Academic outputs (publications/presentations)
- [ ] Understanding and improving clinical practice in Hypoglycaemia care.
- [ ] Quality improvement for local Hypoglycaemia management
- [ ] Requirement to complete an audit as part of training/degree
- [ ] Other

9. Have you achieved these goals by the end of your participation? *

- ○ Yes
- ○ Partially
- ○ No

10. If partially, please specify *

11. If no, please explain why not and what we could do to help you achieve it *

12. Did you use the DEKODE Hypoglycaemia google form to **collect** data? *

- ○ Yes
- ○ No

13. Did you receive training prior to data collection? *

- ◯ Yes
- ◯ No

14. If yes, what type of training did you receive? *

- ◯ In-person
- ◯ Online
- ◯ Both
- ◯ Other

15. If other, please specify *

16. If no, what type of training would have benefitted you? *

17. How did you identify Hypoglycaemia cases? (select all that apply) *

- ☐ List of episodes obtained from IT department
- ☐ Screening of diagnostic codes
- ☐ Screening of discharge letters
- ☐ Keyword search
- ☐ Other

18. How many cases did you collect? (enter an approx if you dont know the exact number) *

The value must be a number

19. What your average time spent per month on data collection for DEKODE Hypoglycaemia? *

- ◯ 0-2 hours
- ◯ 2-4 hours
- ◯ 4-6 hours
- ◯ >6 hours

20. Did you encounter any technical difficulties in obtaining patient records? *

- ◯ Yes
- ◯ No

21. If yes, please specify *

[   ]

22. Did you experience technical difficulties with Google Forms? *

- ◯ Yes
- ◯ No

23. If yes, please specify *

[   ]

24. Did you encounter missing information in patient records? *

- ◯ Yes
- ◯ No

25. If yes, please specify the type of information that was missing or why you were not able to obtain the information *

[   ]

26. Did you know who to contact for help or clarification? *

- ◯ Yes
- ◯ No

27. If no, what would have helped? *

28. How would you rate the clarity of questions on the data collection form? (1 = poor, 10 = excellent) *

1	2	3	4	5	6	7	8	9	10

29. How would you rate the flow and organisation of the data collection form? (1 = poor, 10 = excellent) *

1	2	3	4	5	6	7	8	9	10

30. Does your hospital have a guideline on Hypoglycaemia management? *

- Yes
- No
- Unsure

31. Do you think the guideline requires improvement? *

- Yes
- No

32. If yes, please specify areas for improvement required *

33. Did you implement any interventions to your local hospital on Hypoglycaemia management? *

- Yes
- No

34. If yes, please specify *

35. Rate your agreement with the following statement: My understanding of Hypoglycaemia improved through participation (1 = strongly disagree, 10 = strongly agree) *

1	2	3	4	5	6	7	8	9	10

36. Rate your agreement with the following statement: I've applied the knowledge learnt through my involvement in clinical practice (1 = strongly disagree, 10 = strongly agree) *

1	2	3	4	5	6	7	8	9	10

37. Would you like to be involved in other aspects of DEKODE-Hypoglycaemia? *

- ◯ Yes
- ◯ No

38. If yes, please select areas of interest *

- ☐ Data analysis
- ☐ Publication writing
- ☐ Protocol/guideline development
- ☐ Other

39. Please provide any additional suggestions or comments for improving the project

This content is neither created nor endorsed by Microsoft. The data you submit will be sent to the form owner.

Microsoft Forms
