# Supplementary material for: Implementing a multi-centre hypoglycaemia management surveillance model through a co-designed approach to develop actionable insights in the UK
Source: Front Health Serv. 2026 Sep 24;6:1862035. doi: 10.3389/frhs.2026.1862035 (PMC13646156; doi:10.3389/frhs.2026.1862035)
Supplement: Supplementary file 3 [file Datasheet3.pdf]

ID	Role	Hospital	Approx date of start in D
1	End-user	A	10/1/2023
2	End-user	A, C, D, H	1/1/2024
3	End-user	F	10/1/2024
4	End-user	A	10/1/2023
5	End-user	A	10/2/2023
6	End-user	A, C, D, H	12/1/2024
7	End-user	E	9/9/2024
8	End-user	A	9/1/2024
9	End-user	B, G	10/2/2023
10	End-user	A	10/1/2024
11	End-user	A, F, H	11/1/2023
12	End-user	A, C, D, H	2/6/2024
13	End-user	B	8/1/2024
14	End-user	B, G	7/15/2024
15	End-user	F	7/1/2024
16	End-user	J	11/15/2024
17	End-user	J	11/10/2024
18	Clinical expert	J	4/2/2024
19	Clinical expert	G	9/17/2024
20	Clinical expert	E	11/29/2023
21	Clinical expert	A	3/1/2024

Approx date of end in DI	Role in DEKODE-Hypogly	How did you hear about	Why did you participate
3/17/2025	Audit/QIP registration;Da	DEKODE team ;	Research experience and
3/18/2025	Audit/QIP registration;Da	DEKODE team ;	Research experience and
3/18/2025	Data collection;	Word of mouth from coll	Research experience and
3/18/2025	Audit/QIP registration;Da	DEKODE team ;	Research experience and
3/18/2025	Audit/QIP registration;Da	DEKODE team ;	Research experience and
3/18/2025	Audit/QIP registration;Da	DEKODE team ;Word of r	Research experience and
3/18/2025	Data collection;	Conference ;	Research experience and
3/18/2025	Data collection;	DEKODE team ;Word of r	Research experience and
3/18/2025	Audit/QIP registration;Da	DEKODE team ;Was part	Research experience and
3/18/2025	Audit/QIP registration;Da	DEKODE team ;Word of r	Research experience and
3/18/2025	Audit/QIP registration;Da	Word of mouth from coll	Research experience and
9/30/2024	Data collection;Data anal	SIMBA ;DEKODE team ;	Research experience and
12/14/2024	Data collection;	Word of mouth from coll	Research experience and
3/19/2025	Data collection;	DEKODE team ;	Research experience and
3/19/2025	Data collection;	Word of mouth from coll	Research experience and
3/19/2025	Data collection;Data anal	Word of mouth from coll	Requirement to complet
3/26/2025	Data collection;Data anal	DEKODE team ;Word of r	Requirement to complet
2/4/2025	Audit/QIP registration;Cc	DEKODE team ;	Research experience and
3/25/2025	Data analysis;Audit/QIP r	DEKODE team ;	Understanding and impr
3/25/2025	Consultant Lead;	DEKODE team ;	Academic outputs (public
4/3/2025	In-patient lead;	DEKODE team ;	Understanding and impr

Have you achieved these	If partially, please specif	If no, please explain why	Did you use the DEKODE
Yes			Yes
Partially	Have had the opportunity to develop research exp		Yes
Partially	Data collection audit experience, but no outputs y		Yes
Yes			Yes
Yes			Yes
Yes			Yes
Partially	I haven't submitted any audits or done presentatic		Yes
Yes			Yes
Yes			Yes
Yes			Yes
Partially	Papers not yet published		Yes
Yes			Yes
Partially	I think I would have preferred to get more involvec		Yes
Partially	I have not been able to be continuously collect dat		Yes
Partially	Research experience		Yes
Partially	Project incomplete		Yes
Yes			Yes
Partially	Guideline is not available on the intranet		No
Yes			No
Partially	Work is ongoing and more changes needed to imp		No
Yes			No

Did you receive training	If yes, what type of train	If other, please specify	If no, what type of traini
No			Peer teaching from other
Yes	In-person		
Yes	In-person		
No			guidance from team mer
Yes	In-person		
Yes	Other	Friend trained me after t	Online PowerPoint
Yes	In-person		
Yes	In-person		
Yes	In-person		
Yes	In-person		
Yes	In-person		
Yes	Both		
Yes	In-person		
Yes	In-person		
Yes	Online		
No			N/A - my colleague recei
Yes	In-person		

How did you identify Hy	How many cases did you	What your average time	Did you encounter any t
List of episodes obtained	550	4-6 hours	No
Screening of diagnostic c	180	4-6 hours	No
List of episodes obtained	15	2-4 hours	No
List of episodes obtained	80	4-6 hours	No
List of episodes obtained	150	4-6 hours	Yes
List of episodes obtained	70	4-6 hours	No
List of episodes obtained	100	>6 hours	No
List of episodes obtained	100	2-4 hours	No
List of episodes obtained	170	>6 hours	Yes
List of episodes obtained	500	2-4 hours	No
List of episodes obtained	100	4-6 hours	No
List of episodes obtained	100	4-6 hours	No
List of episodes obtained	40	>6 hours	No
List of episodes obtained	70	0-2 hours	No
List of episodes obtained	130	>6 hours	No
List of episodes obtained	50	4-6 hours	Yes
List of episodes obtained	20	2-4 hours	Yes

If yes, please specify	Did you experience tech	If yes, please specify 2	Did you encounter missi
	No		No
	No		Yes
	No		Yes
	No		Yes
Accounts were disabled f	No		No
	No		No
	No		Yes
	No		No
Wolverhampton patient	No		Yes
	No		Yes
	No		No
	No		Yes
	No		Yes
	No		Yes
	No		No
Paper records - time con:	No		Yes
Paper records at Walsall	No		Yes

If yes, please specify the	Did you know who to co	If no, what would have h	How would you rate the
			10
Exact time of discharge w	Yes		10
Ethnicity	Yes		8
Exact Date/Time of disch	Yes		10
	Yes		10
	Yes		10
Sometimes the exact typ	Yes		10
	Yes		8
Height, weight, HbA1c ar	Yes		10
Certain information can b	Yes		8
	Yes		8
Fluids most often missing	Yes		10
GP records were not link	Yes		7
Many a times hypoglycae	Yes		10
	Yes		10
A huge variety e.g. hypo	Yes		6
Most of the clerking/adm	Yes		8
			9
			8
			9
			10

How would you rate the	Does your hospital have	Do you think the guidelii	If yes, please specify are
10	Yes	Yes	Despite guidelines, patiei
10	Unsure		
6	Unsure		
10	Yes	Yes	Despite clear trust and na
5	Yes	No	
10	Yes	No	
7	Unsure		
8	Yes	No	
9	Yes	No	
8	Yes	No	
8	Yes	No	
9	Yes	No	
8	Yes	No	
10	Unsure		
10	Unsure		
3	Unsure		
1	Unsure		
9	Yes	No	
7	Yes	No	
9	Yes	No	
10	Yes	Yes	Specify level of glucose a

Did you implement any i	If yes, please specify 3	Rate your agreement wi	Rate your agreement wi
No		10	10
No		10	10
No		6	7
No		10	10
No		10	10
Yes	Not as of yet but intervie	10	6
No		8	8
No		7	7
No		9	10
No		8	9
No		10	2
No		8	8
No		8	7
No		10	10
No		10	10
No		4	4
No		8	8
Yes	Hypo box checking regua	9	9
No		8	8
Yes	Improved patient educat	7	7
Yes	Will update guideline	7	8

Would you like to be inv	If yes, please select area	Please provide any addit
Yes	Protocol/guideline devel	None, it has all round been a very useful and insightful expe
Yes	Data analysis ;Publication writing ;Protocol/guideline development ;	
No		
Yes	Data analysis ;Publication writing ;Protocol/guideline development ;	
Yes	Data analysis ;Publication writing ;Protocol/guideline development ;	
Yes	Publication writing ;Protocol/guideline development ;Data analysis ;	
Yes	Data analysis ;Publication writing ;Protocol/guideline development ;	
Yes	Publication writing ;	
Yes	Publication writing ;	
Yes	Publication writing ;Protocol/guideline development ;Data analysis ;	
Yes	Protocol/guideline development ;Publication writing ;Data analysis ;	
Yes	Data analysis ;Publication writing ;Protocol/guideline development ;	
Yes	Publication writing ;	
Yes	Publication writing ;Protocol/guideline development ;Data analysis ;	
Yes	Data analysis ;Publication writing ;	
No		The data collection form needs serious reediting. It needs t
No		Google form question order can be improved
Yes	Publication writing ;Protocol/guideline development ;	
Yes	Publication writing ;Protocol/guideline development ;	
Yes	Protocol/guideline devel	Sharing best practice from different centres
Yes	Protocol/guideline devel	Look at cause of hypos and harm .

o follow a chronological manner and be more concise
