# Supplementary material for: Implementing a multi-centre hypoglycaemia management surveillance model through a co-designed approach to develop actionable insights in the UK
Source: Front Health Serv. 2026 Sep 24;6:1862035. doi: 10.3389/frhs.2026.1862035 (PMC13646156; doi:10.3389/frhs.2026.1862035)
Supplement: Supplementary file 5 [file Table2.docx]

Quarterly Feedback Report, December 2024 for Hospital J from DEKODE Hypoglycaemia study: Numbers given are all numbers of hypoglycaemic episodes in each category.

## IHSG level 2

|  | | All hospitals | Hospital J |
| --- | --- | --- | --- |
| Number of episodes, n | | 931 | 23 |
| Type of Diabetes, n (%) | Type 1 | 312 (33.5) | 3 (13.0) |
|  | Type 2 | 605 (65.0) | 20 (87.0) |
|  | Other | 14 (1.5) | 0 (0.0) |
| Furthest line treatment during the episode, n (%) | Oral glucose | 367 (39.4) | 9 (39.1) |
|  | IV Dextrose | 284 (30.5) | 4 (17.4) |
|  | Glucagon administered | 31 (3.3) | 0 (0) |
|  | Not fully documented | 249 (26.7) | 10 (43.5)) |
| Episode Outcome, n (%) | Admitted to hospital | 58 (6.2) | 0 (0) |
|  | Care continued as inpatient | 794 (85.3) | 23 (100) |
|  | Admitted to ITU | 1 (0.1) | 0 (0) |
|  | Discharged home | 57 (6.1) | 0 (0) |
|  | Death | 21 (2.3) | 0 (0) |
| Outcome on Discharge, n (%)* | Simple discharge | 358 (38.5) | 15 (65.2) |
|  | Complex discharge | 357 (38.3) | 6 (26.1) |
|  | Death | 139 (14.9) | 2 (8.7) |
| Adjustment to therapy, n (%) | Change in Medications | 442 (47.5) | 10 (43.5) |
|  | Specialist team review | 259 (27.8) | 8 (34.8) |
|  | CGM started | 21 (2.3) | 0 (0) |
|  | Glucagon prescribed on discharge | 2 (0.2) | 0 (0) |

## IHSG level 3

|  | | All hospitals | Hospital A |
| --- | --- | --- | --- |
| Number of episodes, n (%) | | 520 | 1 |
| Type of Diabetes, n (%) | Type 1 | 138 (26.5) | 0 (0) |
|  | Type 2 | 381 (73.3) | 1 (100) |
|  | Other | 1 (0.2) | 0 (0) |
| Furthest line treatment during the episode, n (%) | Oral glucose | 88 (16.9) | 0 (0) |
|  | IV Dextrose | 253 (48.7) | 0(0) |
|  | Glucagon administered | 148 (28.5) | 0 (0) |
|  | Not fully documented | 31 (6.0) | 1 (100) |
| Episode Outcome, n (%) | Admitted to hospital | 119 (22.9) | 0 (0) |
|  | Care continued as inpatient | 243 (46.7) | 0 (0) |
|  | Admitted to ITU | 12 (2.3) | 0 (0) |
|  | Discharged home | 91 (17.5) | 0 (0) |
|  | Death | 55 (10.6) | 1 (100) |
| Outcome on Discharge, n (%)* | Simple discharge | 211 (40.6) | 2 (67) |
|  | Complex discharge | 136 (26.2) | 0 (0) |
|  | Death | 138 (26.5) | 1 (100) |
| Adjustment to therapy, n (%) | Change in Medications | 264 (50.8) | 0 (0) |
|  | Specialist team review | 192 (36.9) | 0 (0) |
|  | CGM started | 19 (3.7) | 0 (0) |
|  | Glucagon prescribed on discharge | 7 (1.3) | 0 (0) |

* Complex discharge defined as requiring further care, outpatient follow-up or MDT input. Numbers and percentages do not include discharges other than ‘Simple’, ‘Comple’ or ‘Died’ – these discharges include discharge against medical advice.
