# Supplementary material for: Implementing a multi-centre hypoglycaemia management surveillance model through a co-designed approach to develop actionable insights in the UK
Source: Front Health Serv. 2026 Sep 24;6:1862035. doi: 10.3389/frhs.2026.1862035 (PMC13646156; doi:10.3389/frhs.2026.1862035)
Supplement: Supplementary file 6 [file Table3.docx]

Table 1: Assessment of DEKODE hypoglycaemia model in comparison to the Clinical Audit Quality recommendations from the NICE and HQIP.

| Criteria | Assessment Question | Source | Addressed in the Study |
| --- | --- | --- | --- |
| Audit objectives are clearly defined and aligned with departmental goals and confirming current good practice or improving current practice. | Are the objectives of the audit clearly defined? | Both | Yes, the objectives were clearly defined to audit hypoglycaemia management and identify existing gaps in auditing practices across multiple hospitals. |
|  | Does the objective align with improving or confirming current clinical practices? | HQIP | Yes, the objective was to improve the management of hypoglycaemia by developing a standardised data collection tool and implementing it across multiple hospitals. |
| Involvement of relevant stakeholders, including resident doctors, consultants, and patients. | Are relevant stakeholders engaged in the audit process? | NICE | Yes, stakeholders, including clinical experts, resident doctors, medical students, and resident doctors, were involved in the design, testing, and implementation of the QIP system. |
|  | Is there evidence of stakeholder meeting minutes and attendance records? | NICE | Yes, initial and final stakeholder meetings were held to define the scope, discuss findings, and finalise the data collection tool (mentioned in the co-designing section). |
| Formation of a multidisciplinary audit team. | Has a multidisciplinary audit team been formed with clearly defined roles and responsibilities? | NICE | Yes, a multidisciplinary team was formed, including consultants, resident doctors, and medical students, with defined roles for overseeing processes, data collection, and training. |
| Selection of audit criteria based on the best available evidence and clinical guidelines, reflecting critical aspects of patient care. | Are the audit criteria based on the best available evidence and clinical guidelines? | NICE | Yes, the criteria were based on IHSG Level 2 and Level 3 episodes, aligning with the International Hypoglycaemia Study's guidelines. |
|  | Do the audit standards cover all key aspects of the clinical practice related to the audit’s objective? | HQIP | Yes, the audit standards covered essential areas such as glucose levels, treatment protocols, and patient outcomes. |
|  | Are the criteria relevant to critical aspects of patient care in diabetes and endocrinology? | NICE | Yes, the criteria were relevant to managing hypoglycaemia, a critical aspect of diabetes care. |
|  | Are the measures (standards) sensitive and specific enough to accurately identify true positive and true negative cases? | HQIP | While the focus was on relevant hypoglycaemic episodes, the sensitivity and specificity of the standards are discussed and all steps to increase these values have been taken. |
| Comprehensive and reliable data collection plan, including sources, methods, and quality assurance. | Is there a comprehensive plan for data collection, including sources and methods? | NICE | Yes, the study involved developing a comprehensive data collection tool, a two-tiered data storage system, and training sessions for consistent data entry across hospitals. |
|  | Are key terms defined, and instructions for decision-making specified? | HQIP | Yes, key parameters were defined, and categorical data were simplified to ensure consistent data collection. |
|  | Are data collectors properly trained, and is inter-rater reliability tested to ensure consistency? | Both | Yes, training sessions were conducted for data collectors, and data entry was tested for formatting issues and gaps before full implementation. |
|  | Was data collection pilot-tested before full implementation? | HQIP | Yes, pilot testing was conducted in Hospital A to identify and resolve formatting issues and gaps in the data fields. |
| Data sources are specified and capable of yielding quality data. | Are the data sources for each standard clearly specified? | HQIP | Yes, the data sources included hospital records of blood glucose levels, with specific focus on IHSG Level 2 and Level 3 episodes. |
|  | Are the data sources likely to produce high-quality data efficiently? | HQIP | Yes, the data sources were considered reliable, and the system was designed to ensure efficient data collection across multiple hospitals. |
| Data quality is ensured through validation, accuracy, completeness, and adherence to protocols. | Are the data accurate, complete, and fit for purpose? | Both | Yes, regular validation checks were performed, and any anomalies were reviewed and corrected to ensure data accuracy. |
|  | Is adherence to the case selection and data collection protocol monitored? | HQIP | Yes, adherence was ensured through training, regular validation checks, and monitoring by the audit team. |
|  | Are there processes in place to prevent threats to data quality during collection and collation? | HQIP | Yes, a pseudonymisation system was used, and anomalies were flagged and reviewed to maintain data quality. |
| Development and implementation of an action plan based on audit findings. | Is there a written action plan with timelines and responsibilities? | NICE | Yes, the study included the development of a protocol for implementing the system across hospitals and assigning roles and responsibilities to ensure effective data collection. |
|  | Is there evidence of effective implementation of recommended changes? | NICE | Yes, the data collection system was iteratively refined based on feedback and successfully implemented across ten hospitals. |
|  | Are necessary resources allocated for implementing changes? | NICE | Yes, resources included the involvement of medical students, resident doctors, and consultants, as well as training and support resources for consistent implementation. |
| Continuous monitoring, follow-up audits, and dissemination of results. | Are follow-up audits conducted to ensure sustained improvement? | NICE | Follow-up audits are being planned to assess the impact of interventions. |
|  | Are mechanisms established for continuous monitoring and feedback? | NICE | Yes, regular validation checks and feedback mechanisms were established, including quarterly feedback reports to participating centres. |
|  | Are audit results regularly reported and disseminated to all stakeholders? | NICE | Yes, results were disseminated through structured quarterly feedback reports to stakeholders. |
| Provision of training and access to consultation and support for staff involved in audits. | Are training programs provided for staff involved in audits? | NICE | Yes, training programs were provided for medical students and resident doctors involved in the data collection process. |
|  | Is there access to consultation and support from experienced audit supervisors? | NICE | Yes, the process involved regular team meetings and consultation with clinical experts to guide the audit process. |
| Promotion of a supportive culture and integration of audit activities with clinical governance. | Is there a culture that supports audit activities and continuous improvement? | NICE | Yes, the study created a collaborative culture involving stakeholders at multiple levels, and training ensured that new cohorts could sustain the audit activities. |
|  | Are audit activities integrated with broader clinical governance frameworks? | NICE | Yes, the study’s design and implementation processes align with clinical governance principles, though specific integration details are not explicitly mentioned. |

Table 2: All results by hospital. All results given as n (%), where n represents the number of patients. Age given as median (IQR).

| Hospital |  | Overall | A | B | C | D | E | F | G | H | I | J |
| --- | --- | --- | --- | --- | --- | --- | --- | --- | --- | --- | --- | --- |
| Cohort |  | 945 (100.0) | 205 (21.7) | 63 (6.7) | 119 (12.6) | 123 (13.0) | 255 (27.0) | 142 (15.0) | 7 (0.7) | 18 (1.9) | 1 (0.1) | 12 (1.3) |
| Sex | Female | 451 (47.7) | 85 (41.5) | 38 (60.3) | 52 (43.7) | 66 (53.7) | 129 (50.6) | 64 (45.1) | 5 (71.4) | 6 (33.3) | 0 (0.0) | 6 (50.0) |
|  | Male | 494 (52.3) | 120 (58.5) | 25 (39.7) | 67 (56.3) | 57 (46.3) | 126 (49.4) | 78 (54.9) | 2 (28.6) | 12 (66.7) | 1 (100.0) | 6 (50.0) |
|  | F:M ratio | 1:1.10 | 1:1.41 | 1.52:1 | 1:1.28 | 1.16:1 | 1.02:1 | 1:1.22 | 2.5:1 | 1:2 | N/A | 1:1 |
| Ethnicity* | White | 635 (71.5) | 142 (70.6) | 13 (23.2) | 92 (85.2) | 70 (62.5) | 179 (73.4) | 116 (91.3) | 3 (42.9) | 11 (78.6) | 0 (0.0) | 9 (75.0) |
|  | Asian | 165 (18.6) | 44 (21.9) | 35 (62.5) | 7 (6.5) | 27 (24.1) | 38 (15.6) | 7 (5.5) | 3 (42.9) | 1 (7.1) | 1 (100.0) | 2 (16.7) |
|  | Black | 73 (8.2) | 9 (4.5) | 8 (14.3) | 8 (7.4) | 13 (11.6) | 27 (11.1) | 4 (3.1) | 1 (14.3) | 2 (14.3) | 0 (0.0) | 1 (8.3) |
|  | Other/mixed | 15 (1.7) | 6 (3.0) | 6 (10.7) | 0 (0.0) | 1 (0.9) | 2 (0.8) | 0 (0.0) | 0 (0.0) | 0 (0.0) | 0 (0.0) | 0 (0.0) |
| Age |  | 72 (59-82) | 63 (50-75) | 71 (58-83) | 75 (59-84) | 68 (59-78) | 73 (59-83) | 75 (63-82) | 82 (73-85) | 82 (75-86) | 87 (87-87) | 77 (58-81) |
| Diabetes Type | 1 | 248 (26.2) | 49 (23.9) | 11 (17.5) | 40 (33.6) | 31 (25.2) | 73 (28.6) | 36 (25.4) | 2 (28.6) | 3 (16.7) | 0 (0.0) | 3 (25.0) |
|  | 2 | 686 (72.6) | 151 (73.7) | 52 (82.5) | 78 (65.5) | 89 (72.4) | 180 (70.6) | 106 (74.6) | 5 (71.4) | 15 (83.3) | 1 (100.0) | 9 (75.0) |
|  | Other type | 11 (1.2) | 5 (2.4) | 0 (0.0) | 1 (0.8) | 3 (2.4) | 2 (0.8) | 0 (0.0) | 0 (0.0) | 0 (0.0) | 0 (0.0) | 0 (0.0) |

* Excluding unknowns not listed on hospital records.

Table 3: Comparison of results by episode between Hospitals A and D, two similar centres in the same NHS trust. All results given as n (%), where n represents the number of episodes. Age given as median (IQR). Where p values are not given there was no significant difference.

|  |  | A | D | p |
| --- | --- | --- | --- | --- |
| Cohort |  | 361 | 147 |  |
| Sex | Female | 182 (50.4) | 87 (59.2) |  |
|  | Male | 179 (49.6) | 60 (40.8) |  |
| Ethnicity* | White | 230 (65.2) | 92 (68.1) |  |
|  | Asian | 78 (22.1) | 27 (20.0) |  |
|  | Black | 39 (11.0) | 15 (11.1) |  |
|  | Other/mixed | 6 (1.7) | 1 (0.7) |  |
| Age | Years, Median (IQR) | 63 (50-75) | 68 (59-78) | 0.0118 |
| Diabetes Type | 1 | 115 (31.9) | 42 (28.6) |  |
|  | 2 | 240 (66.5) | 100 (68.0) |  |
|  | Other type | 6 (1.7) | 5 (3.4) |  |
| Antidiabetic medications | Insulin | 272 (75.3) | 98 (66.7) | 0.0485 |
|  | Biguanides | 99 (27.4) | 46 (31.3) |  |
|  | Sulfonylureas | 26 (7.2) | 14 (9.5) |  |
|  | DPP4I | 46 (12.7) | 18 (12.2) |  |
|  | SGLT2 | 21 (5.8) | 5 (3.4) |  |
|  | GLP-1 | 2 (0.6) | 0 (0.0) |  |
|  | Thiazolidinediones | 0 (0.0) | 0 (0.0) |  |
|  | Antipsychotics | 18 (5.0) | 4 (2.7) |  |
| IHSG Event level | 2 | 265 (73.4) | 88 (59.9) | 0.004 |
|  | 3 | 96 (26.6) | 59 (40.1) |  |
| Cognitive impairment* | Yes | 146 (56.8) | 69 (63.9) |  |
|  | No | 111 (43.2) | 39 (36.1) |  |
| MMSE score* | 0-10 | 10 (90.9) | 0 |  |
|  | 10-20 | 1 (9.1) | 0 |  |
|  | 20-35 | 0 (0.0) | 0 |  |
| Precipitant | Fasting/missed meals | 203 (56.2) | 33 (22.4) | <0.0001 |
|  | Intercurrent illness | 113 (31.3) | 96 (65.3) | <0.0001 |
|  | Incorrect medication dose | 31 (8.6) | 11 (7.5) |  |
|  | Exercise | 0 (0.0) | 0 (0.0) |  |
|  | Unclear | 78 (21.6) | 30 (20.4) |  |
|  | Multiple | 57 (15.8) | 18 (12.2) |  |
|  | Single | 304 (84.2) | 129 (87.8) |  |
| Furthest line treatment reached | Not fully documented | 50 (13.9) | 27 (18.4) |  |
|  | Oral glucose | 97 (26.9) | 23 (15.6) | 0.0079 |
|  | IV Dextrose | 198 (54.8) | 87 (59.2) |  |
|  | Glucagon | 16 (4.4) | 10 (6.8) |  |
| Total number of episodes where treatment was used* | Oral glucose | 212 (73.6) | 61 (55.0) | 0.0005 |
|  | Dextrose | 211 (58.6) | 97 (66.0) |  |
|  | Glucagon | 16 (3.4) | 10 (5.7) |  |
| Hyperglycaemia | Yes | 5 (1.4) | 7 (4.8) | 0.0459 |
|  | No | 356 (98.6) | 140 (95.2) |  |
| Episode Outcome | Admitted to hospital | 4 (1.1) | 2 (1.4) |  |
|  | Care continued as inpatient | 332 (92.0) | 120 (81.6) | 0.0015 |
|  | Sent to ITU | 6 (1.7) | 5 (3.4) |  |
|  | Sent home | 1 (0.3) | 1 (0.7) |  |
|  | Death | 18 (5.0) | 19 (12.9) | 0.004 |
| Discharge outcome | Died | 87 (24.1) | 51 (34.7) | 0.0206 |
|  | Complex | 165 (45.7) | 53 (36.1) | 0.0486 |
|  | Simple | 87 (24.1) | 32 (21.8) |  |
|  | Other | 22 (6.1) | 11 (7.5) |  |
| Adjustment | Medication change | 128 (35.5) | 74 (50.3) | 0.0026 |
|  | Specialist team referral | 65 (34.1) | 46 (29.3) | 0.0014 |
|  | Palliative care plan | 86 (23.8) | 30 (20.4) |  |
|  | CGM started | 10 (2.8) | 1 (0.7) |  |
|  | Glucagon prescribed | 1 (0.3) | 0 (0.0) |  |
| Medication change | Stop sulfonylureas | 5 (3.9) | 9 (12.2) | 0.041 |
|  | Reduce sulfonylureas | 7 (5.5) | 3 (4.1) |  |
|  | Stop insulin | 16 (12.5) | 18 (24.3) | 0.0496 |
|  | Reduce insulin | 97 (75.8) | 39 (52.7) | 0.001 |
|  | Changes to others | 24 (18.8) | 18 (24.3) |  |

* Excluding unknowns not listed on hospital records.
